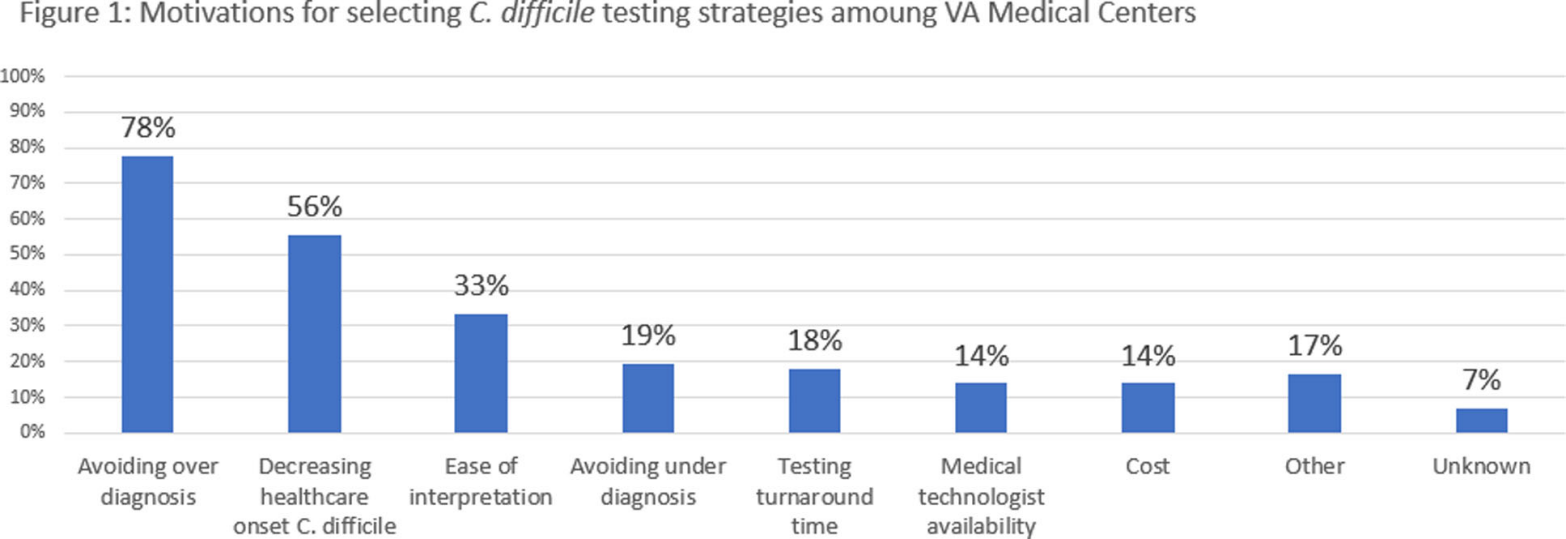# 122 How the COVID-19 Pandemic Reshaped Managerial Skills in IPC Units of Israeli Public Hospitals

**DOI:** 10.1017/ash.2026.10536

**Published:** 2026-06-23

**Authors:** Elise Martin, Robin Jump, Dimitri Drekonja, University of MN Medical School

**Affiliations:** 1 VA Pittsburgh Healthcare System; 2 Minneapolis VA Health Care System

## Abstract

**Background:** There is no gold standard approach to Clostridioides difficile (C. difficile) testing clinically. The National Healthcare Safety Network (NHSN) uses the last test in a series to determine if positive testing will be considered a healthcare associated infection (HAI) and there is concern that this may impact testing choice. Increasingly, healthcare facilities are considering 2-step testing algorithms, but limited data exists on clinical or surveillance motivations for algorithm choice. **Methods:** In October and November 2023, we distributed electronic surveys to Veterans Affairs Medical Center (VAMC) infection preventionists and infectious diseases physicians asking about current facility C. difficile testing practices, motivations for their current strategy, and considerations for change. Only one response was included per facility. Duplicate responses from the same facility were combined. Data were analyzed using Chi-squared tests. **Results:** Among 126 VAMCs, 72 (57%) completed the survey. The most common testing strategies were polymerase chain reaction (PCR) with reflex to toxin (n=31, 43%) and PCR with reflex to glutamate dehydrogenase (GDH)/toxin (n=20, 28%) when PCR is positive. Less common strategies included PCR alone (n=9, 13%), GDH/toxin with reflex to PCR when discordant (either GDH or toxin positive) (n=5, 7%), GDH/toxin alone (n=1, 1%), and other (n=6, 8%). Factors associated with testing strategy selection were avoidance of overdiagnosis (78%), to decrease rate of C. difficile HAIs (56%), and ease of interpretation (33%) (Figure 1). When comparing 2-step testing algorithms, avoiding overdiagnosis was a motivation for positive PCR with reflex to toxin or GDH/toxin (84%) as well as GDH/toxin with reflex to PCR (80%) (p=0.8). When compared to PCR with reflex to toxin or GDH/toxin, sites using GDH/toxin with reflex to PCR more frequently reported test turn-around-time (80% vs 14%, p≤0.001) and cost (60% vs 8%, p≤0.001) as motivating factors. Sites using PCR with reflex to toxin or GDH/toxin were more motivated by decreasing HAIs than GDH/toxin with reflex to PCR (63% vs 40%), although not statistically significant. Notably, 40% of facilities reported switching their testing strategy within 2 years of survey and 24% were actively considering a change. **Conclusion:** The most common testing strategy among VAMCs is a 2-step algorithm starting with PCR, then reflex to either toxin or GDH/toxin when PCR positive, driven by desire to avoid overdiagnosis and decrease HAI rates. It is unclear how this actually impacts rates or if this will change if facilities move to healthcare facility-onset, treated C. difficile infection metrics.

**Figure:**